# Supplementary figures and images for: A hybridoma-derived monoclonal antibody with high homology to the aberrant myeloma light chain
Source: PLoS One. 2021 Oct 11;16(10):e0252558. doi: 10.1371/journal.pone.0252558 (PMC8504763; doi:10.1371/journal.pone.0252558)

Fig 1

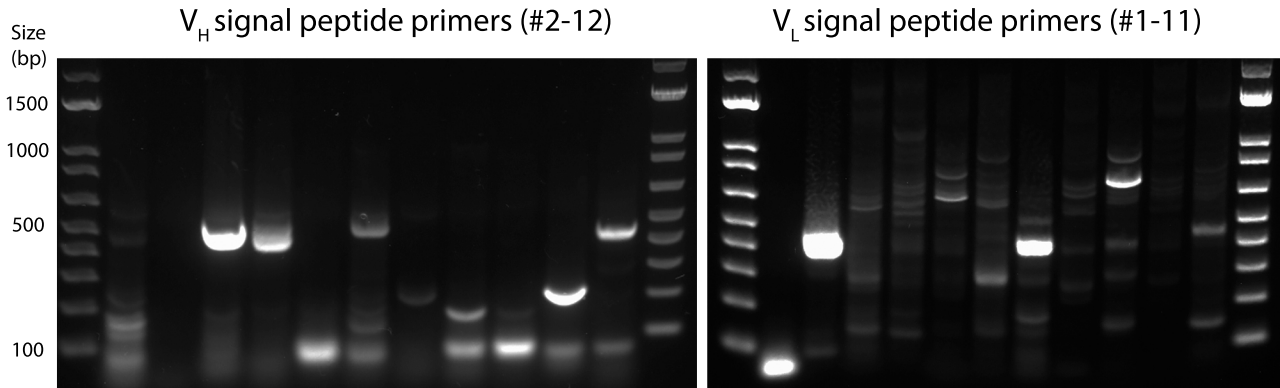

Fig 3B

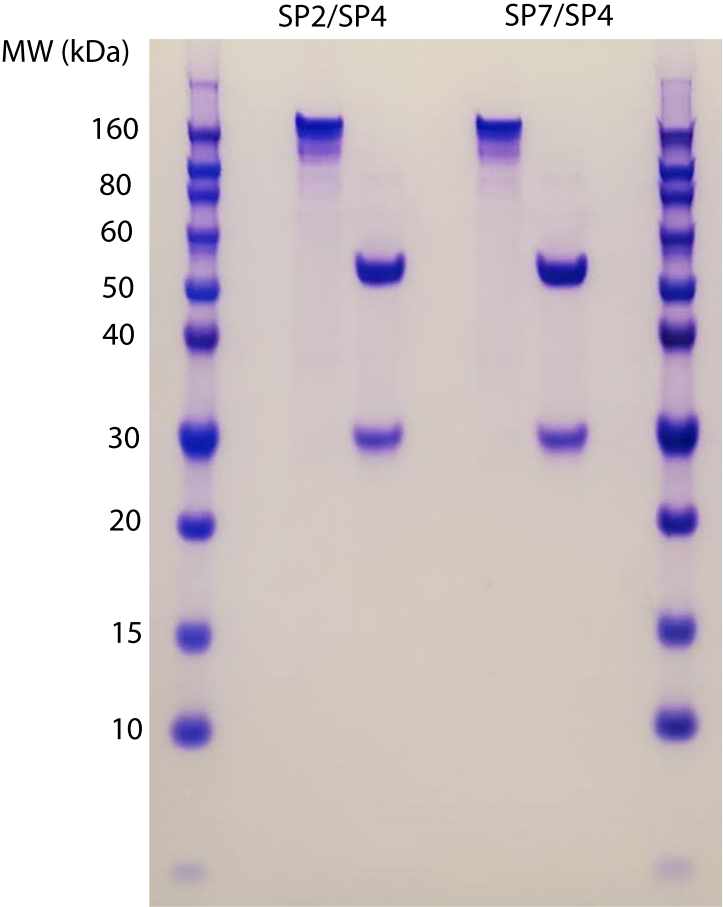

Supplement: S1 File — (PDF) [file pone.0252558.s001.pdf]
