# Supplementary material for: A hybridoma-derived monoclonal antibody with high homology to the aberrant myeloma light chain
Source: PLoS One. 2021 Oct 11;16(10):e0252558. doi: 10.1371/journal.pone.0252558 (PMC8504763; doi:10.1371/journal.pone.0252558)
Supplement: S2 File — (PDF) [file pone.0252558.s002.pdf]

Fig S1

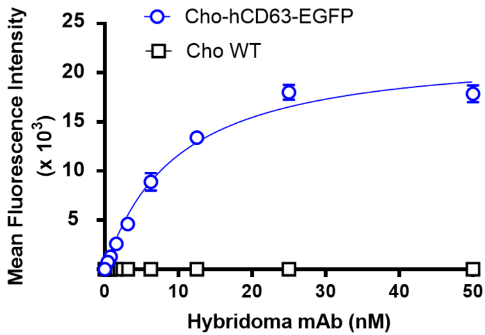

	Mean Fluorescence Intensity		
Concentration (nM)	CHO-hCD63-EGFP		CHO WT
0	13.59	13.68	13.68
0.390625	798.7	692.77	692.77
0.78125	1426.23	1153.5	1153.5
1.5625	2808.89	2385.78	2385.78
3.125	4520.24	4701.53	4701.53
6.25	9543.67	8254.47	8254.47
12.5	13182.37	13619.39	13619.39
25	17470.04	18535.71	17265.7
50	18473.61	17265.7	18535.71

Fig S1. Binding curve of H5C6 mAb to hCD63-EGFP expressing Cho cells and WT Cho cells

Fig S2

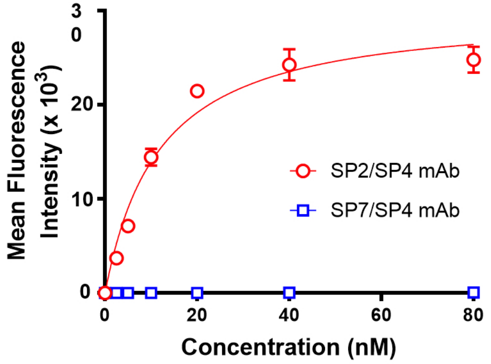

	Mean Fluorescence Intensity			
Concentration (nM)	SP2/SP4 mAb		SP7/SP4 mAb	
80	25757.04	23820.56	45.85	48.48987
40	25419.74	23076.36	30.1	29.83023
20	21077.27	21865.9	22.07	21.35034
10	13789.18	15060.04	20.91	17.61483
5	7283.21	6983.821	17.22	19.27741
2.5	3709.45	3709.07	16.86	15.88974
0	17.38	17.64	16.1	17.04

Fig S2. Binding curve of SP2/SP4 mAb and SP7/SP4 mAb to Cho-hCD63-eGFP cells

Fig S3

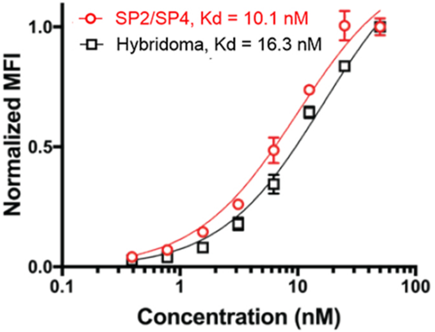

	Mean Fluorescence Intensity							
**Concentration (nM)**	Hybridoma CHO-CD63		Hybridoma CHO WT		Recombinant CHO-CD63		Recombinant CHO WT	
50	1.008191	0.991809	0.001774	0.001958	1.024784	0.975216	0.000664	0.000664
25	0.836719	0.836515	0.001826	0.001994	0.961806	1.047987	0.000514	0.000514
12.5	0.627185	0.660714	0.001758	0.001922	0.742779	0.73203	0.00046	0.00046
6.25	0.317152	0.372107	0.001758	0.001954	0.523746	0.447892	0.000437	0.000437
3.125	0.160862	0.197539	0.001663	0.001899	0.25537	0.265778	0.000447	0.000447
1.5625	0.076719	0.084526	0.001643	0.00181	0.156184	0.13479	0.000436	0.000436
0.78125	0.03914	0.040645	0.001728	0.001915	0.077103	0.064074	0.000398	0.000398
0.390625	0.030952	0.024285	0.001692	0.001902	0.04424	0.03766	0.000427	0.000427
0	0.004837	0.004853	0.001466	0.001649	0.000753	0.000756	0.000257	0.000257

Fig S3. Affinities of SP2/SP4 mAb and H5C6 hybridoma-derived mAb
